# Relationship between care networks and happiness in older immigrants in Australia

**DOI:** 10.1111/ajag.70022

**Published:** 2025-04-28

**Authors:** Shuang Liu, Sunil Bhar, Kumchong Lee, Nancy A. Pachana, Jack Lam

**Affiliations:** ^1^ School of Communication and Arts The University of Queensland Brisbane Queensland Australia; ^2^ Department of Psychological Sciences Swinburne University of Technology Melbourne Victoria Australia; ^3^ School of Psychology The University of Queensland Brisbane Queensland Australia; ^4^ University of Melbourne Melbourne Victoria Australia

**Keywords:** ageing, community care planning, community networks, geriatric health service, happiness

## Abstract

**Objective:**

This study aimed to examine the relationship between care networks and perceived happiness in older immigrants who live in their own homes in Australia.

**Methods:**

A cross‐sectional survey was conducted with 101 participants aged 65–97 years and from seven cultural groups. Participants completed measures of perceived happiness, care network structure, function and adequacy, and demographics. Data were analysed using correlations, analyses of variance and multiple regression analyses.

**Results:**

Family support, giving and receiving emotional and instrumental support and satisfaction with care networks and physical health were significantly correlated with perceived happiness. Regression analyses identified satisfaction with care networks, satisfaction with physical health and receiving instrumental support as significant predictors of happiness, explaining 41% of the variance in happiness.

**Conclusions:**

The findings emphasised the importance of instrumental support from family and formal community aged care services, and satisfaction with care networks to perceived happiness in older immigrants. The study suggested improving communication between older immigrants, their family and service providers to effectively support older immigrants to age well in Australia.

**Practice impact**

Satisfaction with care networks, receiving instrumental support and satisfaction with physical health are important predictors of perceived happiness in older immigrants. Service providers, working with family carers, can shape their practice to improve consumer satisfaction with care networks and widen opportunities for supporting physical health and instrumental care.

## 1 INTRODUCTION

The ageing population is culturally diverse. In Australia, for example, 35% of people aged 65 years or older were overseas‐born and from culturally and linguistically diverse backgrounds.^1^ Both the size and the diversity of the ageing population highlight the need to identify evidence‐based policies and practices for supporting older adults from diverse cultures to live happily outside their homeland.^2^ However, research shows that older immigrants tend to experience lower levels of happiness compared to their local‐born counterparts.^3^, ^4^ Given happiness has been documented to improve health and longevity,^5^ knowledge of predictors of happiness can be translated into aged care services to improve older immigrants' satisfaction with their ageing experiences in their host countries.

The concept of happiness is defined as the perception of subjective well‐being and is sometimes used interchangeably with life satisfaction.^2^ Informed by the convoy model of care in social gerontology,^6^ care networks refer to a set of social relations through which aged care support is provided, received and exchanged.^7^ The convoy model has been used to understand social relations and interactions unique to different care networks, which can vary in structure, function and adequacy.^8^ The structure of a care network includes the size, composition and frequency of contact of people involved in the network. Function refers to the type of care support provided and received.^9^ Adequacy indicates an older adult's satisfaction with their care network.^10^

International research has identified strong positive associations between happiness and receiving family and social support through care networks of older adults. For example, data from 34 countries in Eastern and Southern Europe revealed that older adults living with and receiving care from their daughters were happier than those living alone.^11^ Panagiotopoulos et al.^12^ found that older Greek widows living in Australia identified familial support as a strong positive contributor to subjective well‐being, and lower levels of familial support were related to poor physical health, depression and loneliness. Also, reciprocal care exchanged between adult children and older parents in Chinese immigrant families was found to contribute to the perceived happiness of older Chinese immigrants in Australia.^13^ These findings highlighted the important contribution of the relational aspect of aged care networks to the perceived happiness of older adults, including older immigrants.

Despite recognising aged care as a relational process,^14^ and the importance of family relationships in care networks to happiness,^9^ previous studies have primarily focused on assessing the structure of care networks rather than other components.^15^, ^16^ Limited research has investigated how the three components of care networks (structure, function and adequacy) may predict happiness in older immigrants as they age in their host country. This study aimed to examine the extent to which these components collectively and individually predicted happiness in older immigrants in Australia. The findings have the potential to shape aged care services to more adequately address the needs of older immigrants from diverse cultural backgrounds.

## 2 METHODS

### 2.1 Study design

As part of a larger project, a cross‐sectional survey was conducted to examine the relationship between care network components and perceived happiness of community‐dwelling older immigrants in Australia. The study has received ethics approval from the Human Research Ethics Committee at The University of Queensland (2022/HE000799).

### 2.2 Participants

Purposive sampling strategies were employed to recruit participants with the assistance of several formal community service providers. These providers specialised in delivering in‐home aged care support (e.g. assisting with cooking, cleaning or shopping) and organising regular social activities (e.g. gathering for singing, storytelling or physical exercises) for older clients from diverse cultural backgrounds. They advertised this study in their newsletters, distributed the information sheet (translated by professional translation services in multiple languages) and promoted the study at their clients' social activities. To be eligible for this study, participants needed to meet the criteria: (a) aged 65 years or older, (b) born overseas, (c) currently living in their own home and (d) identified with a culture not predominantly represented in Australia or spoke a language other than English at home (this did not exclude older adults born in South Africa where English is spoken along with their ethnic languages).

The sample comprised 101 participants. Sample characteristics are shown in Table 1. The age of participants ranged from 65 to 97 (mean 77.04, SD 7.46). The sample was predominantly women and residing in Queensland. The average length of residence in Australia was just over 33 years. Nearly half of the sample lived with their partner, while a third lived alone. Participants came from seven cultural groups, with 74% identifying as Asian (e.g. Chinese, Vietnamese and Korean).

**TABLE 1:** Sample characteristics.

Variable	Descriptives
Age, years	
Mean (SD), range	77.04 (7.46), 65–97
Length of stay in Australia, years	
Mean (SD), range	33.50 (15.52), 6–68
Subjective rating of physical health	
Mean (SD), range	3.17 (.96), 1–5
Gender, *n* (%)	
Female	72 (71)
Male	29 (29)
Location of residence in Australia, *n* (%)	
Queensland	79 (78)
Tasmania	20 (20)
Western Australia	1 (1)
Victoria	1 (1)
Current living arrangement, *n* (%)	
Living with partner/spouse but not with children/children's family	49 (49)
Living alone	31 (31)
Living with children/children's family	19 (19)
Other	2 (2)
Cultural group, *n* (%)	
Chinese	24 (24)
Vietnamese	22 (22)
Korean	13 (13)
Other Asian	16 (16)
European	11 (11)
African	8 (8)
Latin American	5 (5)
Oceanian	2 (2)

*Note*: Subjective rating of physical health is measured by a single item ‘How do you rate your physical health? (1 being very unhealthy and 5 being very healthy)’. Other Asian comprises Japanese (*n* = 5), Malaysian (*n* = 3), Lao (*n* = 2), Burman (*n* = 2), Syrian (*n* = 2), Indian (*n* = 1), Pakistani (*n* = 1). European comprises Greek (*n* = 5), Polish (*n* = 2), German (*n* = 1), Spanish (*n* = 1), Serbian (*n* = 1), Hungarian (*n* = 1). African comprises South African (*n* = 5), Angolan (*n* = 2), Sudanese (*n* = 1). Latin American comprises Salvadorian (*n* = 1), Chilian (*n* = 1), Colombian (*n* = 1), Peruvian (*n* = 1), Uruguayan (*n* = 1). Oceanian comprises Fijian (*n* = 2).

### 2.3 Procedure

Informed consent was obtained from each participant who expressed interest in participating in the study and who met the eligibility criteria. The survey was administered to each participant verbally on a one‐on‐one basis by members of the research team, including trained research assistants, in six languages (English, Korean, Mandarin, Cantonese, Spanish and Vietnamese). The research assistants who assisted with administering the questionnaire in other languages were native speakers of the specific language. Prior to the survey, they were provided with training and role‐play practice scenarios by the project team to ensure that they explained the questionnaires accurately to participants in their language. Data were collected between January and May 2023, with 78% face‐to‐face at a participant's home or in a room at a community service provider, and the remaining 22% conducted online through Zoom. The average duration of each survey was 15 min.

### 2.4 Measures

The measures included perceived happiness, care network components (structure, function and adequacy) and demographic variables.

#### 2.4.1 Perceived happiness

One question assessed *perceived happiness* (‘How happy do you feel now?’). Participants were asked to rate this question using a scale from 1 (‘very sad’) to 5 (‘very happy’). A single direct question has been commonly used to assess happiness and has been found reliable and valid.^17^, ^18^

#### 2.4.2 Structure of care networks

Structure of care networks was measured using the Lubben Social Network Scale—6 (LSNS‐6).^19^ Derived from the original 10‐item scale designed to assess social networks and support among older people, the LSNS‐6 comprises six items, with three questions related to family ties (Fam; e.g. ‘How many relatives do you see or hear from at least once a month?’) and three questions on friend ties (Friend: e.g. ‘How many friends do you see or hear from at least once a month?’). The subscale scores for family and friend ties were the sum of item scores within those subscales. In this study, internal consistency alphas for the family ties subscale and friend ties subscale were .779 and .744, respectively.

#### 2.4.3 Function of care networks

Function of care networks was measured with the brief two‐way Social Support Scale (2‐Way SSS).^20^ The original 20‐item SSS was developed to assess types (emotional and instrumental) and directions (giving and receiving) of social support. The 2‐Way SSS is a shortened 12‐item version of the SSS, with three items measuring each of the following subscales: receiving emotional support (RE; e.g. ‘Having someone to lean on when feeling down’), receiving instrumental support (RI; e.g. ‘Having someone to help when feeling physically unwell’), giving emotional support (GE; e.g. ‘People confiding in me when they have problems’) and giving instrumental support (GI; e.g. ‘Helping others when they are unable to fulfil their responsibility’). Item scores within each subscale were added to generate a total score for that subscale. In this study, the internal consistency of the subscales ranged from .684 to .874.

#### 2.4.4 Adequacy of care networks

Adequacy of care networks was measured by a single question about participants' overall satisfaction with their care network as a whole (‘All considered, how satisfied are you with your aged care network as a whole?’ 1—completely dissatisfied; 5—completely satisfied). A single‐item overall care rating was considered a valid measure of patient satisfaction and used to measure satisfaction with aged care.^21^, ^22^

#### 2.4.5 Demographic variables

Demographic variables measured were: gender, age, length of residence in Australia, living arrangements, cultural group and subjective rating of physical health (1—‘very unhealthy’; 5 —‘very healthy’).

## 3 RESULTS

### 3.1 Preliminary analyses of relationships between happiness and predictor variables

The relationships between perceived happiness and demographic variables were examined using one‐way Analysis of Variance (ANOVA; gender and living arrangements) and correlational analyses (age, length of residence and subjective rating of physical health). Perceived happiness was not significantly different across levels of gender [*F*(1, 98) = .30, *p* = .59], living arrangement [*F*(3, 96) = .15, *p* = .93], cultural group [*F*(7, 94) = 1.40, *p* = .21], nor correlated with age, *r* = .06, *p* = .57, or length of stay in Australia, *r* = .05, *p* = .57. Perceived happiness was significantly associated with satisfaction with physical health, *r* = .38, *p* < .001.

Zero‐order correlations were conducted to explore the relationships between perceived happiness and care network structure, function and adequacy. Table 2 shows descriptives and correlation coefficients for these key variables. Higher levels of perceived happiness were significantly correlated with higher scores on the family subscale of LSNS‐6 (Fam), and each of the four subscales of the 2‐WAY SSS and adequacy of care networks.

**TABLE 2:** Descriptives of key variables and correlations with perceived happiness.

Key variables	Descriptives		Perceived happiness^a^	
	Mean	SD	*r*	*p*
Perceived happiness	3.94	.91		
Adequacy	4.16	.94	.48	<.001
Fam	8.53	3.20	.22	.03
Friend	7.54	3.29	.14	.13
RE	10.38	3.98	.45	<.001
RI	10.88	3.92	.44	<.001
GE	9.88	3.63	.30	.003
GI	8.70	4.55	.37	<.001

*Note*: Adequacy = single item measuring overall satisfaction with care networks; Fam = Lubben Social Network Scale‐6 (LSNS‐6) Family ties subscale; Friend = LSNS‐6 Friend ties subscale; GE = brief 2‐way giving emotional support subscale; GI = brief 2‐way giving instrumental support subscale; RE = brief 2‐way receiving emotional support subscale; RI = brief 2‐way receiving instrumental support subscale.

^a^ Correlations between perceived happiness and other variables, *n* = 97–100.

### 3.2 Independent predictors of happiness

A simple multiple regression analysis was conducted to examine which correlates were independent predictors of perceived happiness in older immigrants. Seven correlates identified by the preliminary analyses were regressed onto happiness (Table 3). A priori power analysis was conducted using G*Power version 3.1.9.7 to determine the minimum sample size required to detect a significant *R* ^2^ with seven predictors.^23^ The required sample size to achieve 80% power for detecting a medium effect, at a significance criterion of *α* = .05, was *n* = 103.

**TABLE 3:** Regression coefficients of independent predictors of happiness.

	*B*	SE	*β*	*t*	*p*
(Intercept)	1.346	.426		3.163	.002
GE	−.033	.026	−.130	−1.243	.22
GI	.036	.021	.183	1.730	.09
RE	.042	.027	.187	1.533	.13
RI	.068	.030	.287	2.239	.03
Fam	−.035	.033	−.119	−1.062	.29
Adequacy	.259	.088	.272	2.937	.004
Physical	.205	.084	.222	2.448	.016

*Note*: *R* ^2^ = .41, *F*(7, 86) = 8.37, *p* < .001, *R* = .64. Adequacy = single item measuring overall satisfaction with care networks; *B* = unstandardised regression coefficient; Fam = LSNS‐6 family ties subscale; GI = brief 2‐way giving instrumental support subscale; GE = brief 2‐way giving emotional support subscale; RE = brief 2‐way receiving emotional support subscale; RI = brief 2‐way receiving instrumental support subscale; *β* = standardised regression coefficient.

Abbreviation: SE, standard error of *B*.

Once missing values were accounted for, the sample analysed comprised 94 participants and hence was associated with less than 80% power. However, the overall model was significant, and collectively, the variables explained 41% of variance in happiness (Table 3). When controlling for other predictors in the model, three variables were independent predictors of perceived happiness: satisfaction with overall care networks, receiving instrumental support and self‐rated physical health. Giving instrumental support was marginally significant (*p* = .09).

Exploratory supplementary moderator analyses were conducted to explore whether the effects of predictors on happiness were different across cultural groups. Given the small *n* for each group, the overall sample was grouped as Asian (*n* = 75) or other (*n* = 26). Group and interaction terms comprising the compound of each predictor and group were added to the model along with each of the predictors. None of the interaction terms were significant, indicating that the effects of predictors were not significantly different between Asian and other groups (Table S1). Given that these supplementary moderator analyses were underpowered, the results are preliminary.

## 4 DISCUSSION

Limited research has investigated the extent to which the structure, function and adequacy of care networks predict happiness in older immigrants. This study examined the extent to which each of these components of care networks collectively and uniquely predicted happiness in older immigrants in Australia.

Preliminary correlational analyses were conducted to explore the associations between perceived happiness and structure (family‐based support, friend‐based support), function (receiving and giving emotional and instrumental care), adequacy of care networks (satisfaction with care networks) and demographic characteristics (gender, age, length of stay in Australia and satisfaction with physical health). Happiness levels were also compared across gender, living arrangement and cultural group. Happiness was significantly correlated with family‐based support, giving and receiving emotional and instrumental care and adequacy of care networks. Happiness also correlated significantly with one's satisfaction with physical health.

The finding that family‐based support, not friend‐based support, was statistically associated with happiness may reflect the sample in the study where a large proportion was from Asian cultural backgrounds such as Chinese, Vietnamese and Korean. In those cultures, people tend to rely on family ties as a source of support,^24^ perhaps placing less emphasis on friendship ties. Hence, cultural traditions of family care and filial support in these cultures may explain the emphasis on family ties.^25^ Additionally, migration and ageing can result in reduced or loss of social networks and social connectedness,^26^ and hence, as people get older and move away from childhood friendships or lose friendship ties, they may rely more heavily on family support for aged care and companionship.

These correlates were entered simultaneously into a multiple regression model to explore their collective prediction on perceived happiness as well as to identify predictors that independently predicted happiness. Collectively, these predictors accounted for nearly 50% variance in happiness, suggesting that the seven predictors robustly predict levels of happiness among older immigrants.

However, of the seven predictors, only three independently predicted happiness: adequacy of care networks, receiving instrumental care and satisfaction with physical health. Family‐based support was not retained as a unique predictor, suggesting that once the effects of other predictors were controlled, the independent effect of this variable is no longer significant. Similarly, giving and receiving emotional care and giving instrumental care were not independent predictors.

The finding that perceived happiness was predicted uniquely by one's satisfaction with aged care network is consistent with the previous research on happiness in older adults, reaffirming the importance of satisfactory aged care networks for older immigrants' ageing experiences.^11^, ^27^ Happiness was predicted by the function of care characterised by receiving instrumental care, rather than other functions of care, such as giving or receiving emotional support. This finding reinforces the role community aged care services can play in providing needed instrumental support for older immigrants. The finding that satisfaction with health independently predicted happiness is also consistent with previous studies that have identified health status as a predictor of older adults' happiness.^2^ Older adults who are in good health can live more independently without depending on assistance from others, and such independence may be important to older people's positive ageing experiences.^28^, ^29^

The present study did not find significant differences in the strength of the effect of these predictors for different subgroups (Asian vs. others). While these analyses suggest that the predictors are equally important to happiness across regional groups, they are underpowered and require replication with a larger sample to reach statistical power.

The present study also did not find significant differences in levels of happiness by living arrangements (with a partner, by themselves or with children). Our finding is consistent with some but not all studies. For example, a study on Vietnamese immigrants in the United States showed no significant difference in life satisfaction based on different living arrangements.^30^ Conversely, a study on older Koreans in South Korea indicated that those living with their spouse reported the highest happiness scores, while those living alone reported the lowest levels of happiness.^31^ These inconsistent findings could be attributed to the unique characteristics of the sample across these studies. Factors such as the financial independence of participants, the number of children in the family, the care needs of older person in the sample and the acceptance of extended families in the person's culture may collectively affect the extent to which living arrangement predicts happiness. These variables were not measured in the current study and hence would need to be included in future studies on the relationship between living arrangements and happiness among older immigrants.

This study had two further limitations to be addressed in further research. First, while it is possible to examine the role of care networks cross‐sectionally,^9^ the evolving nature of happiness perceptions of older adults necessitates a longitudinal approach to comprehensively understand the dynamic interplay between care network structure, function, adequacy and happiness. Causality could not be established in the current study; longitudinal studies may provide a better understanding of changes over time influenced by changing priorities, thereby informing the design of services tailored to the care needs of older immigrants. Second, the present study did not assess participants' engagement with social activities. Previous research reported that regular participation in social activities is essential in improving psychological well‐being among older adults of diverse cultural backgrounds^30^; hence, it is unknown whether engagement with such activities is a further important predictor of happiness.

The study results have practical implications for aged care providers, suggesting that such providers could shape their practice to focus on improving consumer satisfaction with aged care networks including community‐based services, to help clients maintain physical health and to widen opportunities for instrumental support. Given that satisfaction with care networks is a predictor of happiness, providers could identify and address aspects of service provision in need of improvement. Aged care providers may also examine opportunities to facilitate medical, exercise and healthy lifestyle practices for older immigrants in order to help maintain physical health and independence in this cohort. Finally, older immigrants may over‐rely on families for instrumental support because of cultural traditions, language barriers and lack of knowledge of the aged care systems in the host country. Our findings call for service providers to facilitate communication between older immigrants and their families so that older immigrants can more easily access instrumental care.

## 5 CONCLUSIONS

This study examined the relationship between care network components and perceived happiness in older immigrants in Australia, a key cohort of the ageing population that has been increasing rapidly but remains under‐studied compared to their non‐immigrant counterparts. This study showed that the most important predictors of perceived happiness in immigrants living in Australia are satisfaction with overall care networks, receiving instrumental support and self‐rated physical health. The study also found that support from family rather than friends is positively associated with happiness. With the ageing population continuing to become culturally diverse, more research needs to be directed to this traditionally disadvantaged and under‐studied population of older immigrants.

## FUNDING INFORMATION

This research project is supported by the Australian Research Council Linkage Grant Scheme (LP210200703; 2022–2025).

## CONFLICT OF INTEREST STATEMENT

No conflicts of interest declared.

## Supporting information


Table S1

## Data Availability

The data that support the findings of this study are available upon request from the corresponding author. The data are not publicly available due to privacy or ethical restrictions.
