# Supplementary material for: Relationship between care networks and happiness in older immigrants in Australia
Source: Australas J Ageing. 2025 Apr 28;44(2):e70022. doi: 10.1111/ajag.70022 (PMC12036953; doi:10.1111/ajag.70022)
Supplement: Supplementary file 1 — Table S1 [file AJAG-44-0-s001.docx]

**Table S1:** Regression Coefficients of Independent Predictors of Happiness for Asian and others.

|  |  |  |  |  |  |  | 95% confidence interval for B | |
| --- | --- | --- | --- | --- | --- | --- | --- | --- |
|  | B | Std Error | β | t | *p* |  | Lower bound | Upper bound |
| (Intercept) | 1.201 | 0.448 |  | 2.680 | 0.009 |  | 0.309 | 2.093 |
| GE | 0.088 | 0.085 | .346 | 1.029 | 0.31 |  | -0.082 | 0.257 |
| GI | -0.048 | 0.071 | -.245 | -.682 | 0.497 |  | -0.189 | 0.093 |
| RE | -0.037 | 0.066 | -.164 | -.561 | 0.58 |  | -0.168 | 0.094 |
| RI | 0.022 | 0.064 | .095 | .353 | 0.73 |  | -0.104 | 0.149 |
| Family | -0.017 | 0.082 | -.058 | -.207 | 0.84 |  | -0.181 | 0.147 |
| Adequacy | 0.453 | 0.204 | .476 | 2.217 | 0.03 |  | 0.046 | 0.860 |
| Physical | 0.214 | 0.252 | .231 | .849 | 0.40 |  | -0.287 | 0.715 |
| Group | -0.431 | 1.552 | -.214 | -.278 | 0.78 |  | -3.522 | 2.659 |
| Int_GE | -0.135 | 0.090 | -.790 | -1.508 | 0.14 |  | -0.314 | 0.043 |
| Int_GI | 0.100 | 0.074 | .603 | 1.359 | 0.18 |  | -0.046 | 0.246 |
| Int_RE | 0.091 | 0.074 | .582 | 1.225 | 0.22 |  | -0.057 | 0.238 |
| Int_RI | 0.073 | 0.074 | .478 | .984 | 0.33 |  | -0.075 | 0.221 |
| Int_FAM | -0.046 | 0.089 | -.228 | -.517 | 0.61 |  | -0.222 | 0.131 |
| Int_Adequacy | -0.243 | 0.224 | -.547 | -1.084 | 0.28 |  | -0.689 | 0.203 |
| Int_Physical | 0.054 | 0.261 | .095 | .206 | 0.84 |  | -0.465 | 0.573 |

**Note.** B = unstandardised regression coefficient; Std Error = standard error of B; β = standardised regression coefficient. R² = .41, F (7, 86) = 8.37, *p* < 0.001, R = .64. Adequacy = Single item measuring overall satisfaction with care networks. Family = LSNS-6 Family ties subscale. GI = Brief 2-way giving instrumental support subscale. GE = Brief 2-way giving emotional support subscale. RE = Brief 2-way receiving emotional support subscale. RI = Brief 2-way receiving instrumental support subscale. ^1^Correlations between perceived happiness and other variables. Group = grouping variable (Asian v others). Int_GE = Interaction of group variable (Asian v others) and GE. Int_GI = Interaction of group variable (Asian v others) and GI. Int_RE = Interaction of group variable (Asian v others) and RE. Int_RI = Interaction of group variable (Asian v others) and RI. Int_FAM = Interaction of group variable (Asian v others) and Family. Int_Adequacy = Interaction of group variable (Asian v others) and Adequacy. Int Physical = Interaction of group variable (Asian v others) and Physical.
